# Supplementary material for: Nanosecond anomaly detection with decision trees and real-time application to exotic Higgs decays
Source: Nat Commun. 2024 Apr 25;15:3527. doi: 10.1038/s41467-024-47704-8 (PMC11045859; doi:10.1038/s41467-024-47704-8)
Supplement: Supplementary file 1 — Supplementary Information [file 41467_2024_47704_MOESM1_ESM.pdf]

Supplementary Information

PITT-PACC-2311-v5-s

Nanosecond anomaly detection with decision trees and real-time application to exotic Higgs decays

S. T. Roche[1,2], Q. Bayer[2], B. T. Carlson[2,3], W. C. Ouligian[2], P. Serhiayenka[2], J. Stelzer[2], and T. M. Hong[2]

[1]School of Medicine, Saint Louis University, Saint Louis, MO, USA
[2]Department of Physics and Astronomy, University of Pittsburgh, Pittsburgh, PA, USA
[3]Department of Physics and Engineering, Westmont College, Santa Barbara, CA, USA

April 2, 2024

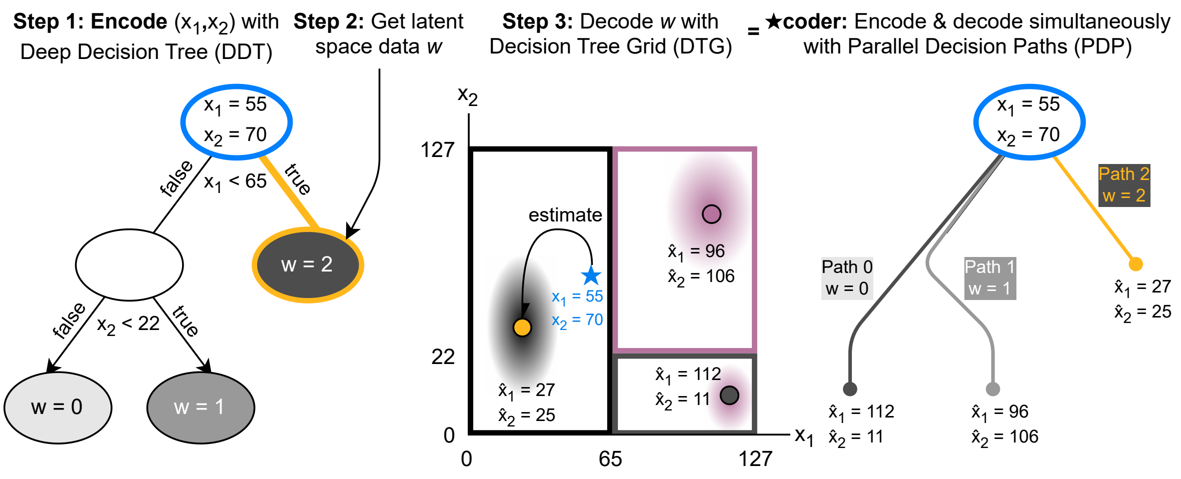

Supplementary Figure 1: Illustrative example of ⋆coder as two visual representations of the same decision tree. Deep decision tree (left) rendered as the decision tree grid (center) and implemented by the parallel decision paths (right). Two-depth deep decision tree (DDT) is the encoder (step 1) shown as a conventional binary split diagram; the latent space is the bin number (step 2); the latent space data is decoded using the decision tree grid (DTG) (step 3); and the simultaneous encoding and decoding with ⋆coder (star-coder) architecture (right) represented by parallel decision paths (PDP) of Ref. [1]. The DTG is the visualization as a grid of partitions in V-dimensional space. In this example, the input $\mathbf{x} = (55, 70)$ yields the output $\hat{\mathbf{x}} = (27, 25)$ without needing to explicitly produce the latent layer.

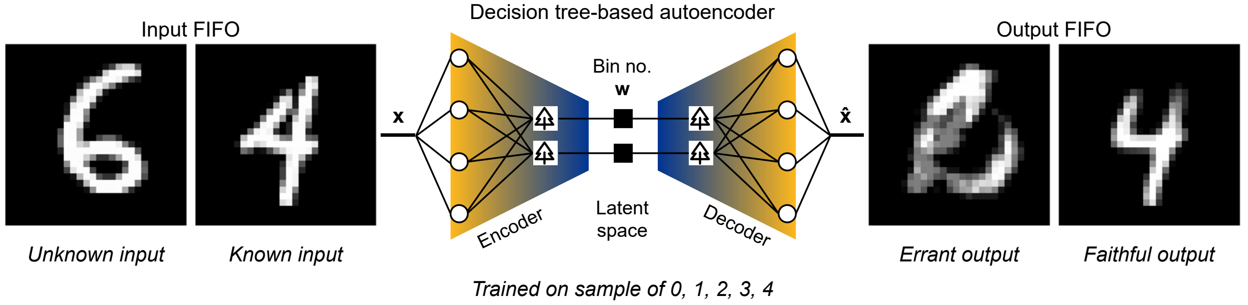

Supplementary Figure 2: Demonstration of decision tree-based autoencoder and a demonstration of data transmission / anomaly detection using the MNIST dataset, which is a set of images of handwritten numbers converted to 28×28 pixels, or 784-length input vector $V = 784$, with $N = 8$ bits per pixel. The ML training is done on 15k images of handwritten 0 to 4, but not 5 to 9, on one tree $T = 1$ at a maximum depth of $D = 20$. The output is a 784-length vector with 8 bits per pixel. The data compression-decompression factor, the ratio of input-output bits to the latent space dimensions, $V \cdot N/(T \cdot D) = 784 \cdot 8/(1 \cdot 20)$, is about 300. The figure shows two input-output pairs as examples. The output of 4 resembles 4 while the output of 6 is garbled. The former yields a smaller input-output distance relative to the latter case. The input data shown here are not part of the training sample.

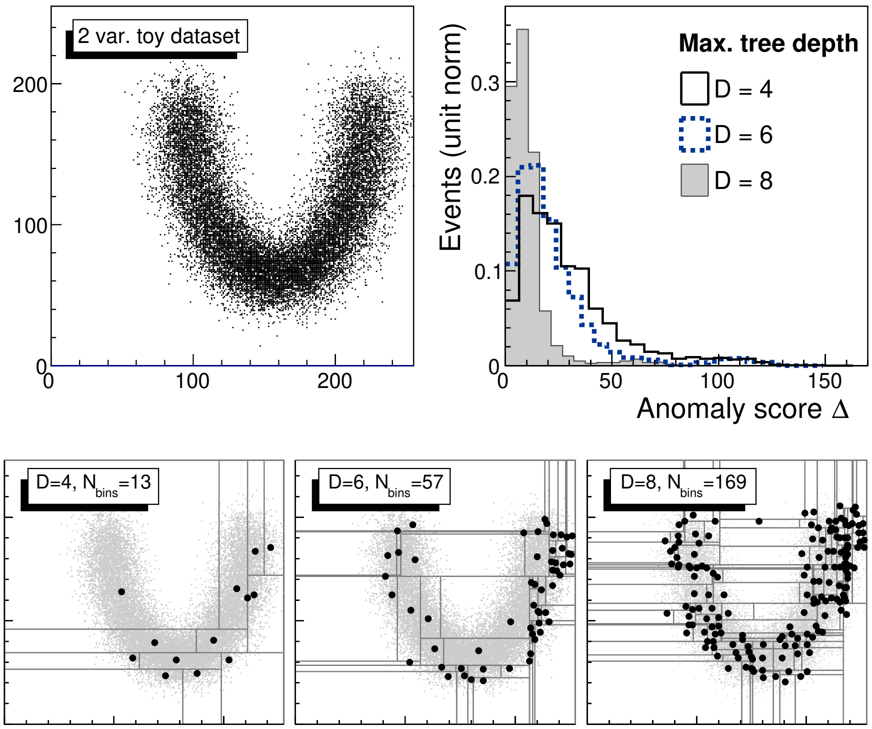

Supplementary Figure 3: Toy dataset and ML training with varying maximum depth D. The top-left plot shows training sample where each data point is represented by a 2d coordinate. The top-right plot shows input-output distance Δ for various D. The anomaly score distribution shows RMS shrinking with D when evaluated on a sample similar to the training sample. The bottom rows of plots shows the result of the ML training. In each partition, a dot (●) indicates the estimate $\hat{\mathbf{x}}$, the location of the median in each dimension of the data in that bin, corresponding to the bin that $\mathbf{x}$ resides in. With the median points one can visualize the refinement of the reconstruction of the original dataset with increasing D.

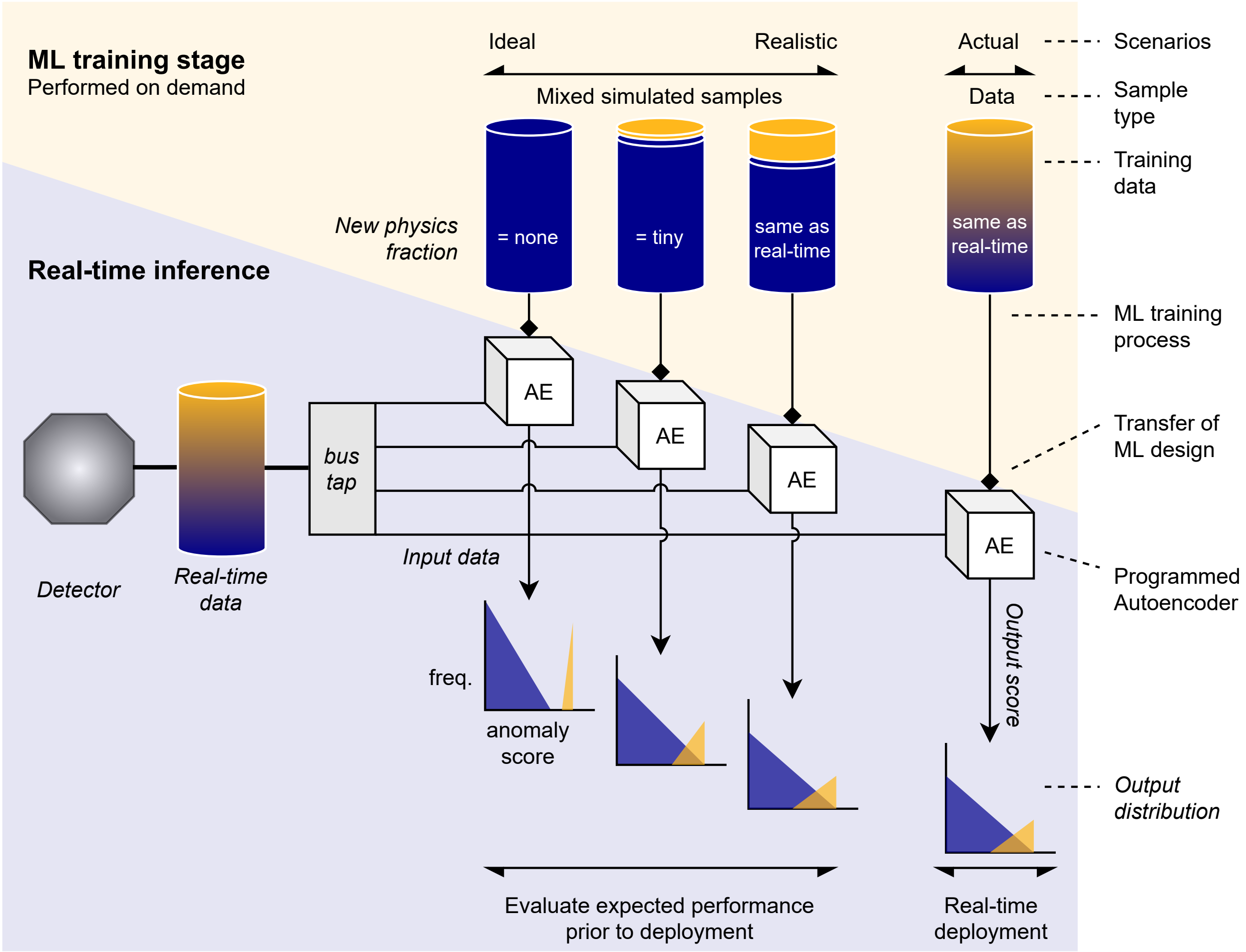

Supplementary Figure 4: Illustration of the ML training with varying levels of signal contamination (top) and the real-time inference (bottom). This setup can help prepare the scenario where the autoencoder is trained using the incoming data itself.

References

[1] B.T. Carlson, Q. Bayer, T.M. Hong, and S.T. Roche, *Nanosecond machine learning regression with deep boosted decision trees in FPGA for high energy physics*, J. Instrum. **17**, P09039 (2022).
